# A Discussion of the Use of Virtual Reality for Training Healthcare Practitioners to Recognize Child Protection Issues

**DOI:** 10.3389/fpubh.2019.00255

**Published:** 2019-09-13

**Authors:** Olivia Drewett, Gayle Hann, Marco Gillies, Carmel Sher, Sylvie Delacroix, Xueni Pan, Tara Collingwoode-Williams, Caroline Fertleman

**Affiliations:** ^1^UCL Medical School, London, United Kingdom; ^2^North Middlesex University Hospital, London, United Kingdom; ^3^Department of Computing, Goldsmiths, University of London, London, United Kingdom; ^4^Birmingham Law School, University of Birmingham, Birmingham, United Kingdom; ^5^Whittington Health, London, United Kingdom; ^6^UCL Institute of Child Health, UCL, London, United Kingdom

**Keywords:** immersive virtual reality, medical training, general practice, medical consultation, child safeguarding

## Abstract

**Background:** Virtual reality technology is a rapidly developing tool which has been shown to have exciting prospects in the field of medical education (1). In a recent, subsequent study, Pan et al. consider the potential of the same technology in the realm of child protection training and safeguarding issues (2). To build upon the Pan et al. (2) study, a panel discussion was held at The Centre for Behavior Change Annual Conference 2018 to discuss the question “Can a virtual reality communication scenario be used to teach General Practitioners and trainees how to recognize and manage child protection issues?.”

**Methodology:** The above study comprised an immersive virtual reality consultation, in which the ability of 63 doctors to pick up covert safeguarding cues was tested in the context of a consultation with an adult patient, where the patient's child happened to be present as well. The study and its findings were discussed at the Centre for Behavior Change 4th Annual Conference, and this paper summarizes the opinions of both the panel and the audience.

**Viewpoint:** Safeguarding is a challenging area of practice where we must listen to the child, and tackle difficult conversations with parents. Within medical training, role play is the gold standard for teaching how to communicate in difficult scenarios. Given the ethical questions surrounding children being asked to role play such abuse, the use of virtual reality characters could have a key role in upgrading current practices in medical education on safeguarding.

## Background

### Virtual Reality Background

Immersive Virtual reality is a technology that creates an illusion for the individual of being physically present in a specific environment, and requires the following:

- Surround graphics: whereby participants cannot see the outside world and the virtual world is visible as they turn their head
- A stereoscopic display: with different images displayed on each eye to simulate stereoscopic vision
- Ambisonic audio: 3D surround sound
- Head tracking: involving the participant's head being tracked so that the view updates as they look around

**Figure 1:**
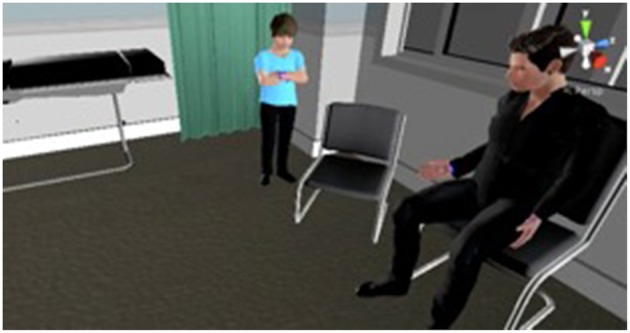
This image demonstrates the virtual characters that were used in the study.

Virtual reality is a powerful tool to reproduce realistic social interactions (3, 4). It is possible to interact with 3D images of people, represented either by a real person interacting live (an avatar) or, as was used in this study, a computer controlled character (an agent). The virtual agents are life size and share the same virtual space as the participant (Figure 1), creating a far more real experience than that achieved through viewing the same environment on screen.

This immersive reality technology has been demonstrated to be an exciting educational tool in clinical consultations through Pan et al.'s study “The Responses of Medical General Practitioners to Unreasonable Patient Demand for Antibiotics—A Study of Medical Ethics Using Immersive Virtual Reality” (1). Pan et al. (2) builds on this earlier work and investigates another scenario in which virtual reality technology could prove useful.

### Child Protection Background

In 2016/17 the NSPCC helpline responded to its highest ever number of calls, and in 2016 there were over 50,000 children in the UK identified as needing protection from abuse (5). These statistics demonstrate the need for professionals from all backgrounds to be vigilant in identifying and safeguarding vulnerable children. Given General Practitioners' (GPs) knowledge of their patients' families and local communities, they are ideally placed to recognize these children and escalate issues when required.

*Outcomes for graduates* is a General Medical Council (GMC) publication which outlines the knowledge, skills and behaviors that UK medical graduates must be able to demonstrate. The section on safeguarding specifies that new graduates must be able to “Identify the signs that suggest children or other vulnerable people may be suffering from abuse or neglect and know what action to take to safeguard their welfare” (6).

The Royal College of General Practitioners builds on this advice, stating that GPs need to be able to act as an advocate for the child in knowing when and how to share concerns about a child who they think might be at risk (7).

Child protection is now an important part of the child health curriculum at medical school (8). Currently, it is primarily taught through lecture material and the use of Serious Case Reviews as examples, together with students' involvement in discussions at multi-disciplinary team (MDT) meetings within the hospital environment. As exposure depends on the current patients and the willingness of the team to engage students, additional practical teaching is needed to demonstrate how subtle these situations can be in real clinical practice. Furthermore, as the teaching is often labeled as “safeguarding training” subtle cues are somewhat expected, which is not reflective of real clinical practice.

Medical students and junior doctors are often taught how to communicate in difficult settings through consultations with actors, to enable mistakes to be made in a setting with no negative consequences for patients and their families. Given the moral questions surrounding the recruitment of children to pretend they have suffered sexual or physical abuse, different teaching methods are required.

While the use of virtual reality in medical education could be extended further, to train receptionists in how to interact with difficult patients for example, such scenarios may be too generic as to have real value, and those that are too specific are limited in their application across multi-disciplinary teams.

### Study Background

After being granted ethical approval, 63 GPs and trainees from local GP practices were recruited for the Pan et al. (2) study and told that they were carrying out a virtual consultation to test new virtual reality software. However, the true objective of the study was to assess the doctors' ability to pick up on subtle safeguarding cues during a virtual consultation with a parent. The responses of the virtual reality agents were pre-programmed, with a researcher selecting the most appropriate response during the consultation based on the questions asked by the doctors.

The consultation required the participants to discuss the pros and cons of mitral valve replacement via the trans-femoral (key hole) route vs. sternotomy (open heart surgery). The patient Chris was accompanied by his 6 year old son Tom who, during the most emotionally intense part of the consultation, interrupts and seeks permission to go to the toilet. Chris refuses to let Tom go, and then later in the consultation answers a phone call on his mobile and leaves the GP and Tom alone for 1 min.

After the consultation the GPs typed up their notes electronically, as they would normally. The GPs were invited to observe their performance and comment on how they felt they dealt with this difficult scenario. The consultations and subsequent notes were analyzed by a team of experts to assess how well the GPs managed the safeguarding concern.

The Pan et al. study aimed to answer two questions about whether the degree of professional experience of the GPs and the cognitive load of the GPs would affect their ability to identify and act upon child safeguarding concerns effectively. Beyond these specific research questions, it was felt that there were other interesting issues in relation to child safeguarding training that were worth considering. Therefore, at the panel discussion the following questions were addressed:

- Can we create a virtual reality consultation with both obvious and more subtle safeguarding cues?
- Can a virtual reality role play scenario be used to enhance safeguarding skills?

In the Pan et al. study, 73% of participants identified the safeguarding element. This demonstrates that identification was possible and indicates the potential of immersive virtual reality as a training tool. However, it also highlights a need for further training to improve this recognition rate. The different approaches taken for interacting with the child also demonstrate a wide range of skill and confidence among the participants in engaging with children.

## Methodology

### Overall Study Methodology

This paper builds upon the findings of the Pan et al. (2) study to focus more specifically on the child safeguarding training dimensions. Before reading this paper, we encourage those interested to look at the original study to better understand the aims and methods of the research. This paper however, analyses a panel discussion which was held at the Centre for Behavior Change 4^th^ Annual Conference at University College London on 21 February 2018.

**Figure 2:**
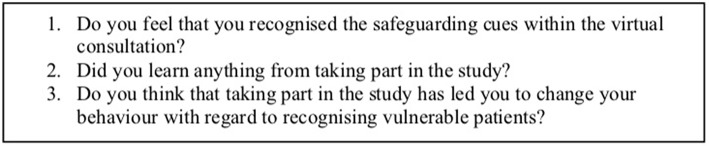
This box contains the questions included in our follow-up questionnaire.

One year on from the Pan et al. data collection, after being granted ethical approval, the original study participants were sent a follow-up questionnaire (Figure 2). The aim of this follow up was to provide qualitative data about whether the GPs felt their inclusion in the study had enabled them to better recognize children at risk.

**Figure 3:**
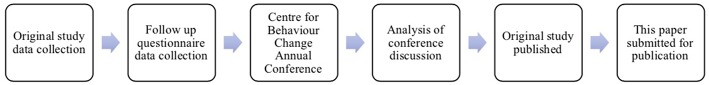
This graphic demonstrates the timeline of the study events.

The conference discussion used both the original study and the new data from the follow-up questionnaire to debate the usefulness of virtual reality in safeguarding training (Figure 3).

### Panel Discussion Methodology

The panel session started with a video which demonstrated the virtual reality consultations (https://sites.google.com/site/panxueni/gpcave). The conference attendees saw how the GPs explained medical treatments to the virtual patient and saw examples of the father's worrying behavior that we hoped the GPs would pick up on—interestingly, nobody in the audience commented on the child not being allowed to go to the toilet.

The audience was asked to watch the video, and to consider the moment that they started to feel uncomfortable. There were various responses to this question, with many pointing to the body language of the child and the father's choice of language during the consultation. While in some ways these observations were cued by the video clip, the discussion that followed did touch on all the aspects of the consultation that the audience was concerned about.

The panel facilitator then led a discussion on the study. The discussion started with an opening statement from each panel member regarding their expert area within the study, and then questions and comments were invited from among the conference attendees.

The panel membership was as follows:

1. Caroline Fertleman: Pediatrician and medical education expert
2. Gayle Hann: Pediatrician and named doctor for child protection
3. Carmel Sher: General Practitioner and study participant
4. Marco Gillies: Virtual reality expert
5. Olivia Drewett: Medical student and Research Assistant on the study

The audience consisted of 70 professionals from the pharmaceutical industry, the charity sector, health psychologists, ethicists, medical doctors, academics, computer scientists, and many others who were passionate about the use of new technology to influence behavioral change. There were conference attendees from over 20 countries, which provided for a varied discussion.

The audience members were encouraged to ask questions and challenge the panel members on their views regarding the use of virtual reality technology in safeguarding training. During the discussion panel member Olivia Drewett wrote notes on the questions asked, responses given and debates that followed. These notes were later re-read and organized into the themes presented below using open coding to clearly summarize the varied discussion.

The authors of this paper recognize that this method is not free from bias however several different viewpoints were expressed and, based on these, this paper aims to present a critical analysis of the use of virtual reality in both safeguarding training and more widely in medical education.

### Emerging Themes

#### Reasons for Differing Responses

The cues may have seemed obvious to a room of conference attendees who knew the panel discussion was about safeguarding. However, those running the study did get to see examples of the “invisible child” through those consultations where the participants did not pick up on the overt child protection cues and took no opportunity to speak to the child alone. The concept of the “invisible child” has been raised in serious case reviews where professionals have missed cases of serious abuse because they have allowed parents' agendas to prevent the consultation being sufficiently child-centered (9).

In this context, the following verbatim quotes from Pan et al. study participants demonstrate how they were also distracted by other elements of the consultation, leading them to miss the safeguarding cues;

“*I focussed too much on the clinical details of the case (explaining the cardiac procedure to the patient) and subsequently missed the safeguarding concern.”*

“*The patient's cardiac problem was hard enough to deal with, leave alone any potential safeguarding issues.”*

“*It was a useful reminder that child protection issues can be part of every consultation, and that confusing factors like complicated medical history can act to distract practitioners from those issues.”*

Carmel Sher suggested that the differences in response to the safeguarding cues depend on a combination of situational and personal (or individual) risk. The situational risk has been standardized between the participants here by using virtual reality, and such standardization is important in using this technology to assess GPs. However, personal risk depends on personal experience, and is multifactorial therefore much harder to standardize. It can be affected by things such as missing a cue in a similar case previously, or having experienced something in one's personal life that has influenced where that person sets the threshold for referral. It relies on the Practitioner's self-awareness, insight, ability to reflect on previous situations and capacity to understand their own thresholds in new situations.

One interesting observation was made by a member of the audience from a health advertising agency in America, who was concerned about whether doctors understood the importance of safeguarding training. The panel argued that due to the scale of child abuse across the world, the NHS and other health authorities have declared that “safeguarding is everyone's business” (10), and the members of the panel had not witnessed a lack of enthusiasm for safeguarding training in the UK. Notwithstanding this, the NSPCC does caution against comparing the rates of child abuse within different countries given varying cultural attitudes, ages of majority, and civil and criminal legislation (11).

#### Cost

Members of the audience from charities were unsure about the cost of the study, and questioned whether the cost of the technology would prohibit it being used more widely in the future. The panel confirmed that the main costs are in the time required to set up the scenario, and for the facilitator who is required to select the responses of the various virtual reality characters.

#### GP Training

The participants in the study thought that they had been recruited to test out new virtual reality equipment, with the safeguarding element not disclosed to them in order to get the most out of the study. For those who did pick up on the covert cues, the consultation was a good opportunity to practice how they would react, think about what they would say to the child and consider what they would write in their notes afterwards. For those who didn't recognize the safeguarding concern, they had a chance to reflect on the consultation afterwards and think about how they would act in a similar consultation in the future.

One study participant said that she was “*angry and upset”* about being misled by the study recruitment, a point which was of interest to one conference attendee with an interest in deception in psychology. This led to a long conversation at the panel discussion about the GP who felt misled.

In the study follow up this GP commented that “*I was very distracted by my thoughts on 'pros and cons of virtual reality vs. role play consultations for student teaching', which is what I thought the study was about. I was thinking about the algorithms for the automated responses, and how realistic it was etc. and not about my own consultation skills.”* Although this participant missed the safeguarding cues during the consultation, they confirmed that the taking part in the study had led them to change their behavior regarding recognizing vulnerable patients. The conference attendees at the panel discussion felt this provided good evidence as to the answer to the final question posed in the aims of the study.

## Viewpoint

As noted above, training consultations about child protection have been recognized as an area where the use of virtual reality agents or avatars could be better than using actors (12). In addition, there is specific research to suggest that it is inappropriate to use children to act out certain scenarios (13).

Prior to conducting the study, there was some concern that the consultation would not feel real, and this may lead doctors to act differently to how they would in clinical practice. Quotes from some of the GPs who took part in the study indicate differing views on this issue.

“*Impressed, evoked a sense of discomfort within me which is difficult to do in an artificial setting.”*

“*Some of the nuances of real human interaction difficult to replicate in this way, especially eye contact.”*

There was much discussion about these quotes at the Behavior Change conference. The audience, who had differing experiences with virtual reality software, were interested in discussing how “real” the consultation felt. The panel acknowledged that the GPs who took part have differing views, but felt that the attention to detail in the design of the virtual consultation room was critical to making it feel “real.”

The panel and audience discussion ended by talking about the fact that although the study did not assess behavior change directly, quotes from GPs who took part demonstrated reflection which will lead to improved actions. One doctor mentioned immediately after finishing the study that they would like the chance to try again with the consultation as they had realized where they had got it wrong.

The panel explained to the audience that it is hoped that, those GPs who missed the safeguarding cues will be far more vigilant in the future to recognizing vulnerable children in their clinics. Ninety percentage of study participants felt they learned about a variety of topics from virtual reality software and its potential application in medical education to their skills in recognizing safeguarding cues and how they should deal with a consultation like this in the future, with one participant commenting:

“*I've never experienced a similar occurrence in real life before, so it was good to have a practice of what you would say to the child to get him to open up to you.”*

## Conclusion

In opening the conference the keynote speaker, John Dinsmore, challenged the attendees to think about some key questions when considering a project such as this, specifically, “Why am I bothering with this project?,” “Is it a national priority?” and “Is there a solution already available?.”

The importance of safeguarding and child protection as an issue to be addressed is apparent through the sheer numbers of vulnerable children in the UK alone. This study sought to build upon the results of an empirical virtual reality study (2) to discuss ways in which the current medical training methods used in the area of child safeguarding may be improved. While the study considered the use of virtual reality to teach GPs only about safeguarding cues, it is recognized that it could be used in similar scenarios on a larger scale to solve other problems with current teaching methods.

Participants in the Pan et al. (2) study confirmed that more realistic (and therefore more expensive) avatars/agents would be required to improve the effectiveness of the virtual reality training tool, and acknowledged this could limit the technology being used more widely. However, in recent years the cost of virtual reality technology has reduced significantly and, as this study and the discussion at the CBC conference confirmed, as a proof of concept at least, its use in safeguarding training provides an exciting prospect for the future.

## Ethics Statement

This study was carried out in accordance with the recommendations of UCL Research Ethics Committee with written informed consent from all subjects. All subjects gave written informed consent in accordance with the Declaration of Helsinki. The protocol was approved by the UCL Research Ethics Committee. Informed consent was obtained from all participants prior to the original study (A Study of Professional Awareness Using Immersive Virtual Reality: The Responses of General Practitioners to Child Safeguarding Concerns, June 2018). This included information about potential side effects and data protection measures. For this paper, only a simple follow up questionnaire sent to the original participants was used. Participants received an email explaining that participation would be voluntary and explaining what the results would be used for. No vulnerable populations were involved.

## Author Contributions

CF facilitated the workshop. OD was on the panel at the workshop and organized the writing up of this research. GH, MG, and CS were on the panel at the workshop to provide expert opinions from their relevant backgrounds. SD was the instigator of the original project, in which XP and TC-W undertook the virtual reality.

### Conflict of Interest Statement

The authors declare that the research was conducted in the absence of any commercial or financial relationships that could be construed as a potential conflict of interest.
